# Wide-angle color holographic near eye display with full bandwidth frequency multiplexing

**DOI:** 10.1038/s41598-025-98411-3

**Published:** 2025-04-30

**Authors:** Maksymilian Chlipala, Maria-Luisa Cruz, Juan Martinez-Carranza, Moncy Idicula, Rafal Kukolowicz, Tomasz Kozacki

**Affiliations:** 1 Faculty of Mechatronics, Institute of Micromechanics and Photonics, Warsaw University of Technology, Warsaw, Poland; 2 Engineering Faculty, Universidad Panamericana, Guadalajara, Mexico

**Keywords:** Imaging and sensing, Computational science

## Abstract

Wide-angle holographic near-eye displays (HNEDs) face challenges in achieving high frame rates, full-color, high-resolution images, and shallow depth of field. Existing full-color methods either rely on time multiplexing or suffer from reduced image resolution due to the applied filtering of the available spatial bandwidth of the light modulator. To address these limitations, we propose a non-pupil HNED system that reconstructs large 3D full-color scenes from a single hologram using RGB LED illumination. The non-pupil architecture allows employing an incoherent LED source, which offers reduced speckle noise and high imaging resolution. This is, to our knowledge, the first wide-angle 3D color holographic display to use a single spatial light modulator (SLM) at full frame rate. Our system is supported by a developed computer generation hologram (CGH) method, which maximizes the SLM’s bandwidth for color coding and improves the speed of RGB CGH calculations. Additionally, we introduce a frequency filter design to simplify color coding and illumination alignment. Through optical reconstructions, we demonstrate that our method preserves 3D depth and color distribution, achieving high-resolution color holographic video without compromising refresh rate or hologram bandwidth.

**Supplementary Information:**

The online version contains supplementary material available at 10.1038/s41598-025-98411-3.

## Introduction

Holographic near-eye displays (HNED) are one of the most promising solutions for displaying three-dimensional images in virtual and augmented reality applications^1^. As holographic displays, the HNEDs reconstruct 3D images with an unlimited number of focus planes, avoiding the vergence-accommodation conflict. However, a genuine 3D immersion experience requires that an HNED produces images with a wide-angle (WA) field of view (FoV)^2,3^, fast frame rate^4^, and full-color^5^. Besides this, the scene high resolution and low speckle noise are strongly desirable. Developing an HNED that includes all these features is still a defiant challenge. For example, the FoV of holographic displays is restricted by the optical imaging system and the Spatial Light Modulator (SLM), where the hologram is displayed^6,7^. Also, the hologram projection plane affects the FoV. In this regard, optical systems for HNEDs can be classified into pupil and non-pupil architectures^8,9^. In pupil HNED, the hologram is projected in the eye-box plane^9^ using a 4 F system that demagnifies the pixel size. The non-pupil HNED projects the hologram in a plane away from the eye-box and the display is composed of the 4 F system and an additional magnifying lens^10,11^.

Besides the FoV enlargement in HNED, displaying full-color images is critical for rich immersion. Full-color HNED has been achieved using three SLMs^12^, color time multiplexing^10,11,13–17^, rainbow holographic setups^18,19^, depth division^20^, and physical color filters by using frequency multiplexing color method (FMCM)^21^. Nevertheless, these methods present some drawbacks. Using three SLMs makes the optical system complex and expensive. Additionally, color time multiplexing requires SLMs with very high frame rates^11^ to avoid unwanted visual effects. Alongside, in rainbow holographic setups, image duplicates of false color are generated up and down the desired image, restricting the eye-box size. The depth division method requires SLM with extended phase depth. Lastly, in the case of physical color filters with FMCM, the three-color components have to be assigned to non-overlapped regions, in the frequency domain of the hologram. This process is achieved by adding a carrier wave^21–23^ or applying the frequency-division method (FDM) in the Fourier domain^24,25^. Despite these limitations, the methods where RGB color encoding and reconstruction are based on spatial filters^16,22^ and color filters^21^ have an important advantage; they combine three RGB component holograms in a single frame, enabling color imaging at full frame rate. Even so, these solutions are characterized by a small FoV. Another important feature in HNED is the use of the full bandwidth of the SLM, which is especially important for wide FoV displays; it defines the trade-off between the field of view and the image resolution. If the image resolution decreases, the depth of field (DOF) increases and limits the number of focus planes on the display, deteriorating the 3D effect. In summary, to the present day, several proposals exist to increase the FoV in HNED^7,10–12,15,26,27^, but only three of them achieve full-color displays, and all of them use color time multiplexing^10,11,15^. Thus, having full-color WA-HNED with the full frame rate is impossible. To solve this challenge, the wise use of the hologram space bandwidth product (SBP) is required to incorporate the three-color channels in a single frame without sacrificing the available frequencies of the SLM. Thus, splitting the SBP for separate colors will minimally reduce the image resolution and depth resolution of the 3D RGB display. This optimal SBP preservation provides high resolution image.

This work proposes a WA-HNED system in the non-pupil configuration capable of reconstructing a large 3D full-color scene from a single frame using RGB LED illumination. To the best of our knowledge, it is the first wide-angle color 3D holographic display using a single SLM at a full frame rate. The proposed color method is a single frame; thus, SBP is divided between color components. The display is supported by the proposed fast and accurate RGB CGH, which is based on a spatial summation of bandwidth-adjusted monochromatic CGHs, and it uses the concept proposed in Ref^7^. However, here, it is shown that the RGB component holograms are calculated using the same sub-hologram, improving RGB CGH’s calculation efficiency. When combining the FMCM^21^ with the non-paraxial RGB CGH calculation, we enable full SLM bandwidth usage for color coding without sacrificing the object bandwidth, thus the proposed method has high resolution. For this purpose, a method for optimizing the bandwidth of RGB channels is proposed. The method finds the optimal values of RGB illumination angles and the position and size of the applied frequency cutoff filter. Only when the available bandwidth for RGB CGH is large does the DOF of the system guarantee the focusing effects when reconstructing 3D objects in color. This high bandwidth is particularly important at high FoV, when the image is very large and consequently has lower resolution. Moreover, it turns out that bandwidth optimization improves the speed of RGB CGH calculations, because for a single point of an RGB object, the hologram is calculated as a sum of very small sub-holograms. The system does not require extra optical elements for the color blend. Nevertheless, a strong relationship exists between the color coding and the illumination angle. A frequency filter design method is developed, making the filter alignment easier. The proposed method allows coding each color component in a different spatial frequency range of the SLM bandwidth. Thus, SLM is illuminated with RGB light sources at different angles on the display. By tilting the illumination of R and G components, the three frequency band gaps overlap in the frequency space, and they are filtered with one filter; all the reconstruction beams propagate on-axis. We propose a simple calibration procedure that ensures full bandwidth transfer of RGB components and is easy to perform. The numerical and optical reconstructions show that the depth and color distribution of the 3D object are preserved. Showed visualization proves that the proposed display can display color holographic videos in high resolution without reducing the frame rate.

## Results

### Display

This paper introduces a full-color WA-HNED, in which RGB LED sources simultaneously illuminate the SLM. It enables the reconstruction of a full-color 3D object without requiring extra optical components for color blending and with a full refresh rate of the SLM. The color is reproduced from a single holographic frame at the full refresh rate of the SLM, using the entire hologram bandwidth. The HNED is designed in a non-pupil configuration, where the hologram is positioned far from the eyebox and relatively near the reconstructed object. Such architecture is advantageous for incoherent illumination because it reduces the speckle noise while maintaining imaging resolution^7,28,29^.

**Fig. 1:**
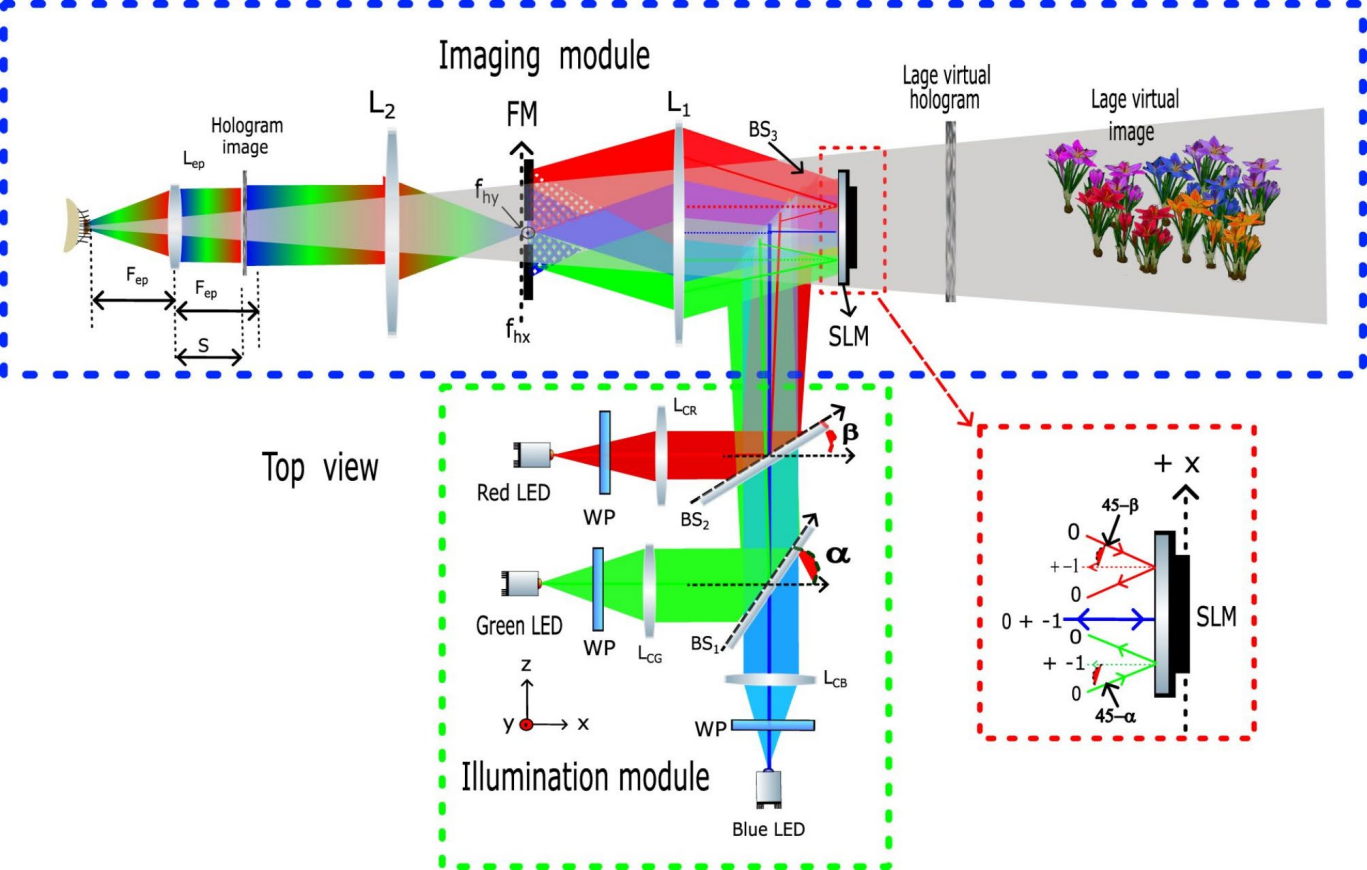
Scheme of the optical setup of full-color HNED with RGB LED sources. *WP* wave plate, *BS* beam splitter, *LC* collimating lens, *L* lens, *FM* filter mask, *SLM* Spatial Light Modulator.

Figure 1 presents the scheme of the full-color HNED developed in this work. The system is divided into two modules: illumination and imaging. The role of the first module, the green rectangle in Fig. 1, is to produce RGB illumination of the SLM, while the second one, the blue rectangle, provides a reconstruction of a large 3D color object. In the illumination module as the light sources, we employ three LED sources (DoricLenses pigtailed LEDs with fiber core size 960 μm) red (*λ*_*R*_ = 635 nm, full width at half maximum Δ*λ*_*R*_ = 20 nm), green (*λ*_*G*_ = 515 nm, Δ*λ*_*G*_ = 35 nm), and blue (*λ*_*B*_ = 465 nm, Δ*λ*_*B*_ = 25 nm). Each beam emerging from the fiber is collimated with the corresponding collimating lens L_CR_ (*f’*_*cR*_ = 300 mm), L_CB_ (*f’*_*cB*_ = 300 mm), and L_CG_ (*f’*_*cG*_ = 300 mm). In our solution, phase-only LCoS (Liquid Crystals of Silicon) SLM (HoloEye Gaea 2.0, pixel count 3840 × 2160, pixel size Δ_SLM_ = 3.74 μm) is illuminated simultaneously by all color components producing white light. However, each color hits the SLM at a different angle along the x-axis; the red rectangle in Fig. 1. The blue beam travels along the z/optical axis and illuminates the SLM at a 0° angle relative to the *z*-axis, while the green and red beams are tilted at -2.8113° and + 3.2811°, respectively. These values are found in the Methods section. To achieve these specific angles, beam-splitters BS_1_ and BS_2_ are adjusted to angles *α* = 47.8113° and *β* = 41.7189°, with respect to the *x*-axis (see Fig. 1), following the method described in the Calibration section. Additionally, the polarization of the RGB components is fine-tuned using three half-wave plates to match the main polarization axis of the liquid crystals in the modulator. The imaging module consists of three lenses. Lenses L_1_ (*f’*_1_ = 100) and L_2_ (*f’*_2_ = 122) form a 4 F imaging system that produces a real, magnified image of the SLM in the back focal plane of lens L_2_. The setup magnification, m_1_, is determined by the ratio of the focal lengths of the lenses, *f’*_2_/*f’*_1_, which equals 1.22. The SLM is then viewed through an eyepiece, L_ep_ (with focal length *f’*_*eye*_ = 33 mm), which acts as a magnifying glass. This setup creates a large virtual image of the hologram that appears far from the observer. The magnification introduced by the eyepiece depends on the distance *s* between the eyepiece and the SLM image. When *s* is close to *f’*_*eye*_, the magnification is large. In our display, *s* is set to 31 mm, resulting in an eyepiece magnification, *m*_*eye*_, of 16.48 and a total display magnification, *m*_*tot*_, of 20.11. Consequently, the pixel size in the hologram plane, Δ, is 75.21 μm, and the physical dimensions of the hologram are 288.80 × 162.45 mm^2^, yielding a maximum FoV of 31.6°.

A crucial component of the optical system is a cut-off filter positioned in the Fourier plane of the SLM. This filter serves two primary functions: first, it decodes complex information encoded in the phase-only hologram, and second, it allows only specific frequencies corresponding to each color object component to pass through. In our display, RGB beams illuminate the SLM at different angles along the *x*-direction, causing the zero-order positions for each color component to be located at different points along the *x*-axis (see filter plane illustrated in Fig. 1). The blue component aligns with the axis, the red component is shifted to the right, and the green component is shifted to the left along the *x*-axis. However, the CGH displayed on the SLM is designed so that the bandwidths used for RGB object coding overlap in the Fourier space for all color components. This alignment allows color imaging from three RGB sources and a single SLM panel.

The display is supported by a CGH algorithm that calculates the RGB hologram using almost full SLM bandwidth. Each RGB channel occupies a frequency band of different size and position. They are frequency-separated in the x-direction, but it should be noted that they are located directly adjacent to each other. The limits of these bands are determined in the Methods section. Their bandwidths *B*_*fx*_×*B*_*fy*_ are given by 0.074 μm^−1^ × 0.097 μm^−1^ for red, 0.091 μm^−1^ × 0.12 μm^−1^ for green, and 0.102 μm^−1^ × 0.133 μm^−1^ for blue. The efficiency of the generated CGH is particularly high in the x-direction, where the CGH occupies the full SLM bandwidth. For this direction, the CGH for the red, blue, and green channels occupies 27.76%, 37.9% and 34.22% of the bandwidth, respectively.

### Experimental results

**Fig. 2:**
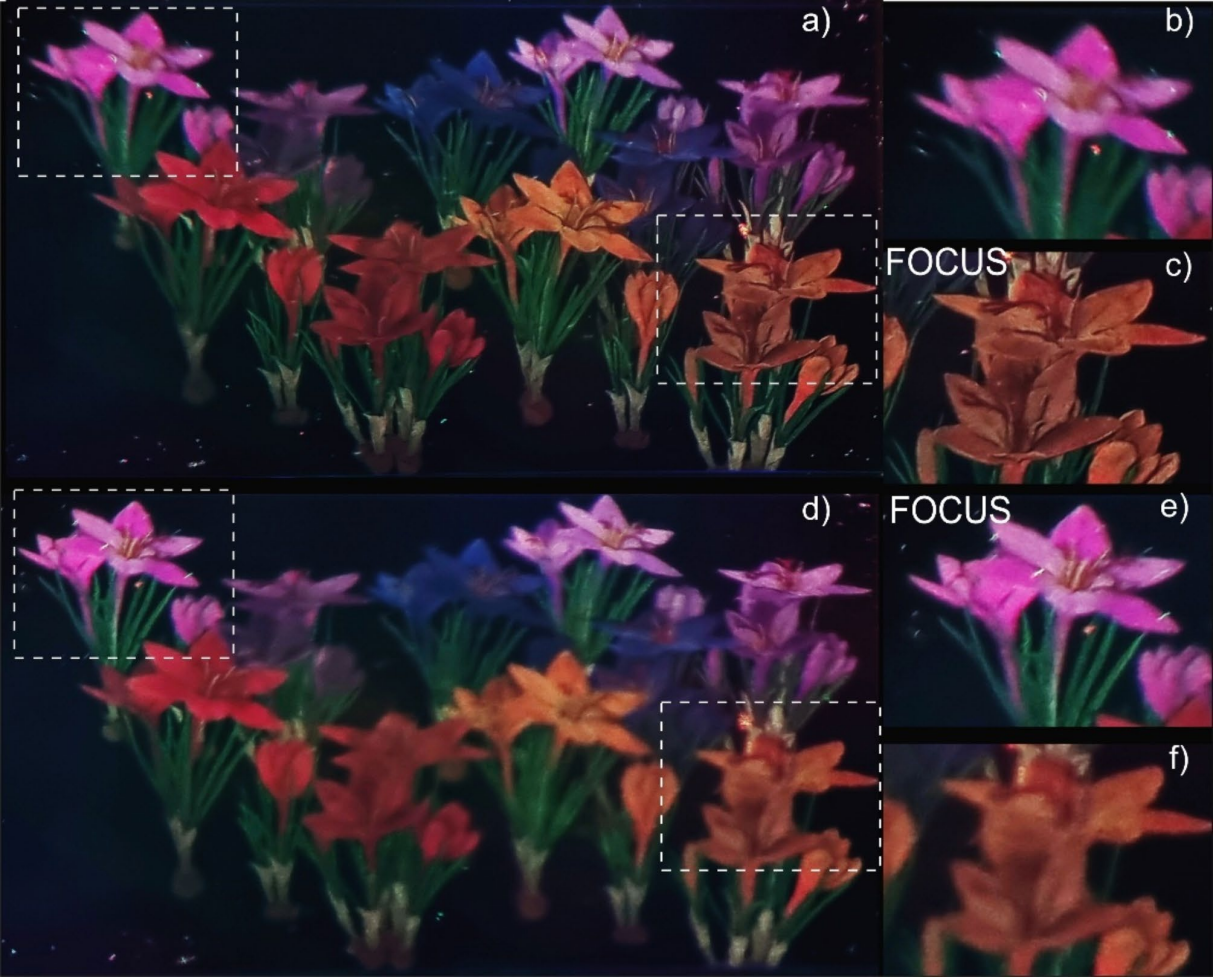
Optical reconstruction of a 3D model of flowers captured at two different focus locations: (**a**–**c**) front and (**d**–**f**) back.

The effectiveness of the proposed method is evaluated through two experiments. The first experiment confirms the system ability to achieve high-quality 3D color object reconstruction, while the second demonstrates its capability to display video content at the full-frame refresh rate of the SLM. In both experiments, the same test object is utilized—a 3D model of colorful flowers, as shown in Fig. 2. The model consists of 20 M points, with each point defined by three spatial coordinates XYZ and three-color components RGB. After occlusion culling the cloud has 2.9 million points^30^. For this cloud density the calculation time for RGB CGH is 111s. The object dimensions are as follows: 430 mm (width) × 270 mm (height) × and 320 mm (depth). The yellow flowers at the bottom right are the closest to the viewer (830 mm), while the violet flowers at the top left are the farthest (1140 mm). The three-color components are combined to generate a single hologram using the method detailed in Section Methods. The results were captured using a smartphone camera positioned in the eyebox plane of the display, allowing easy adjustment of the image focus plane. In the first experiment, static images were recorded. The optical reconstructions captured at two different focus distances are shown in Fig. 2a, d. Two areas of each image are enlarged to provide a more detailed view. In Figs. 2a–c, the focus is on the yellow flowers in the foreground. The details of the yellow flowers, such as the filaments and edges, are sharply defined, while the violet flowers in the background appear blurred. In Figs. 2d–f, the focus is shifted to the violet flowers in the background, bringing them into sharp focus and blurring the yellow flowers. The flowers on the right and left sides of the image show similar clarity to those in the center, as seen in Fig. 2c, e. During the recording of all the images the same hologram was displayed. These results demonstrate that the proposed solution can produce detailed color reconstructions of large and deep 3D objects from a single hologram frame. This capability allows the display of holographic video at the full SLM frame rate, as tested in the second experiment. A sequence of 1140 frames, each showing different views of the flowers, is displayed at the full-frame refresh rate of the SLM, which remains unaffected by the color reconstruction method. In our display, SLM displays 4 K images at a frequency of 58 Hz. The reconstructed images are recorded in real-time, as shown in Supplementary Video 1. The camera is focused on the center of the scene throughout the video. These experimental results validate that one hologram frame generated with our method reconstructs a full-color 3D object with wide-angle, large DOF, with no-speckle noise and high quality. The method uses a simple optical system that is easy to align and is capable of displaying a real dynamic 3D object using the maximum SLM frame refresh rate.

## Discussion

In this work, a full-color WA-HNED is reported. This novel display enables color holographic imaging of large 3D scenes of outstanding quality with full use of SLM bandwidth, at full frame rate, and from a single phase-only hologram. The proposed display leverages a fast, accurate RGB CGH technique. By integrating FMCM with a non-paraxial RGB CGH approach, the system maximizes the SLM bandwidth for color representation, without compromising the object’s spatial bandwidth. When the available bandwidth for RGB CGH is sufficiently broad, the system’s DOF can maintain precise focus, which is essential for reconstructing detailed 3D color objects. Such high bandwidth is particularly crucial when working with a WA-FoV, where larger images inherently suffer from reduced resolution. An interesting feature of the proposed method is that the size of a sub-hologram is the same for all RGB channels, which enhances the calculation efficiency for RGB CGH by 18%.

The solution achieves color blending without requiring additional color filters. However, an important element of the system is cut-off filter. In our work a frequency filter design method has been developed to simplify filter alignment. This approach encodes each color (RGB) within different spatial frequency regions of the SLM bandwidth. The SLM is illuminated by RGB light sources from distinct angles. By tilting the illumination for the red and green components, the frequency bands for all three colors are aligned in the frequency space, allowing them to be filtered with a single filter and ensuring that all reconstruction beams propagate along the optical axis. Optical reconstructions confirm that the 3D object’s depth and color distribution are maintained. Obtained results demonstrate that the presented display allows the delivery of high-resolution color holographic videos without compromising the frame refresh. We compare the solution developed in this work against four HNEDs systems^11,16,18,22^. Those systems were selected because they enable full-color holographic reconstruction of 3D objects. The parameters chosen for comparison are transversal resolution (Δx), efficient frequency bandwidth (EFB = ((frequency color size)/(1/Δx) × 100%), %), color display method, and eyebox size. This work defines frequency color size as the number of pixels employed for calculating the CGH divided by the frequency sampling. The values of these parameters are presented in Table 1.

**Table 1:** Comparison of color HNED systems.

	Δx [µm]	FBE (*R*)	FBE (B)	FBE (G)	FoV	Method	Eyebox (mm) V x H
CAM HNED* [25]	97.35	4.7%	4.7%	4.7%	4.1°	FMCM	7.5 × 7.5
Binocular HNED* [21]	17.57	13.8%	13.8%	13.8%	1.91°	FMCM	No info
WL HNED* [18]	180.8	1.2%	1.2%	1.2%	17.41	FMCM	0.6 × 13
Color HNED*^,++^ [11]	52.34	100%	100%	100%	50°	TM	6.7 × 6.7
This work	47.23	34%	38%	28%	31.6°	FMCM	1.3 × 2.5

*Calculated for green light. ^++^Ideal case.

In the first row of Table 1, HNED system based on pupil configuration is presented^8,25^. This system employs RGB coherent sources, which illuminate the SLM. Moreover, the complex amplitude modulation (CAM)^25^ filter approach is employed, and the channels are multiplexed in the vertical direction. The transversal resolution of system one is almost 100 μm. This is because the applied frequency filter is small such that only 5% of the EFB can be used. The Binocular HNED (Table 1, second row)enables a larger use of the EFB than the previous system, which is around 15%. This configuration allows for an increase in transversal resolution by a factor of five, but the FoV is extremely limited. The binocular HNED employs the frequency superposition multiplexing (FSM) method for obtaining color images. It is worth noting that these two systems have small FoV because the native pixel size of the SLM is preserved. The WL HNED (Table 1, third row)system employs white light illumination and the FSM method to generate the color image. The FoV is around 18°, which is much larger than previous systems. Here, the employed EFB is around 1% percent, which is reflected in a poor transversal resolution (around Δx = 180 μm). In the fourth row, the presented HNED has FoV = 50°, and color features are obtained by time multiplexing. In that system, RGB images are displayed sequentially in the SLM, which decreases the display frame rate. It is worth noting that, the time multiplexing technique allows, in principle, using 100% of the EFB. Nevertheless, in practice, an amplitude filter is necessary for removing high diffraction orders, which decreases the EFB and, therefore, the transversal resolution. Finally, our method is presented in the last row of Table 1. Notably, the EFB in our solution allows us to use, on average, more than 30% of the available EFB, and thus, transversal resolution is better than that of the other systems. The FoV of our system is smaller than the one in Reference^11^ but it can be improved using a 4f system with larger magnification or an eyepiece with smaller focal length. In our system, color features are obtained by the FMCM method, which has the advantage of refreshing the color images at the maximum displaying rate of the SLM. Finally, the last column shows the size of the eyebox of the presented systems. For the first system, the eyebox is given by 7.5 mm x 7.5 mm in both directions. In system two, there is no available information; for the third system, the eye box is a small horizontal slit. The eyebox in the fourth system is square-shaped at 6.7 mm x 6.7 mm. However, it is important to note that the eyebox of the display is smaller because there is amplitude filter conjugated with the eyebox. In our system, we have a rectangular shaped eyebox. This is because the filter size in the x direction is designed to pass one-third of the spectrum while for the y direction, half.

## Methods

### Bandwidth maximization in FMCM

The HNED system described in the Display section aims to generate high-quality 3D color images. For this purpose, it is important to ensure bandwidth maximization of FMCM and full bandwidth transfer in the display. The first can be done when fitting the RGB channels within the full frequency bandwidth of the SLM for the x-direction and passing the maximum bandwidth for the y-direction. Hence, it is necessary to accommodate each color channel into specific frequency bands of the SLM. These RGB bands must be designed considering that the spatial width in the x-directions at the filter plane is the same for all colors. This section finds the minimum and maximum frequencies of these RGB bands. These frequencies should be designed by selecting cut-off frequencies according to the vertical and horizontal dimensions of the 4 F filter of the maximum size.

**Fig. 3:**
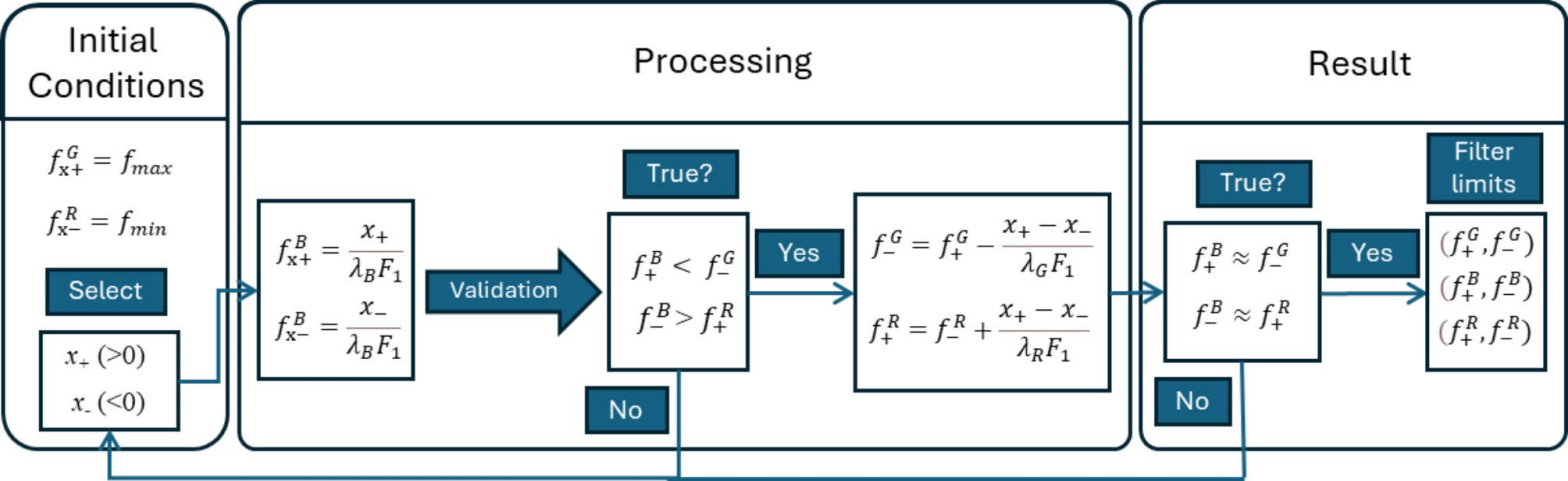
Diagram for frequency limits calculation of RGB bands for *x*-direction.

Boundary frequencies for y-direction are selected according to the complex amplitude method (CAM)^31^. In CAM, a pure cosine grating is employed to convert a complex hologram into a phase-only hologram, which is uploaded into the SLM. This work employs the most effective solution for the bandwidth transferred through the system. It approximately halves the bandwidth in the *y*-direction. The lower edge of the filter corresponds to a frequency close to zero, i.e., *f*_*y-*_ ≈ 0, so it enables the transmission of as many low frequencies as possible while blocking out zero order. On the other hand, the upper edge of the filter is adjusted to the maximum frequency for blue wavelength *f*_*max*_ = 1/2Δ, hence *y*_+_ = *f*_*max*_*λ*^*B*^*F*_*1*_. This value gives the corresponding boundary frequencies of the RGB channels in y-direction as *f*_*y+*_^*B*^ *= f*_*max*_, *f*_*y+*_^*G*^ *= y*_*+*_*/F*_*1*_*λ*^*G*^, and *f*_*y+*_^*R*^ *= y*_*+*_*/F*_*1*_*λ*^*R*^. For the display parameters, the corresponding values are *f*_*y+*_^*R, G,B*^ = [0.097, 0.12, 0.133] µm^−1^. These frequency limits are passed to the 4 F filter calibration procedure (see Fig. 3) and to the FMCM CGH algorithm.

For *x*-direction, RGB holograms occupy different frequency bands within the bandwidth of the SLM, i.e., from *f*_*min*_ = −1/2Δ to *f*_*max*_ *=* 1/2Δ. The algorithm for calculating the frequency limits of RGB bands is shown in the diagram in Fig. 3.

The first block of the diagram in Fig. 3 defines the global maximum and minimum frequency limits, which are *f*_*x+*_^*G*^ = *f*_*max*_ and *f*_*x-*_^*R*^ = *f*_*min*_. Accordingly, the blue channel is located in the central frequency area, red occupies the negative frequencies, and green occupies the positive frequencies. To determine the frequency limits of the blue channel, any two spatial positions *x*_*+*_ (> 0) and *x*_-_ (< 0) are selected, which are the lower and upper edges of the 4 F spatial filter. These coordinates are related to the maximum and minimum frequency of the blue channel as follows

1 $$\:{f}_{\text{x}+}^{B}=\frac{{x}_{+}}{{\lambda\:}_{B}{F}_{1}},$$

2 $$\:{f}_{\text{x}-}^{B}=\frac{{x}_{-}}{{\lambda\:}_{B}{F}_{1}}.$$

These frequencies must satisfy the constraint $\:{f}_{+}^{B}<\:{f}_{-}^{G}$ and $\:{f}_{-}^{B}>\:{f}_{+}^{R}$, otherwise choose different values of *x*_+_ and *x*_–_, as shown in the second block of the diagram. When the constraints are met, the minimum frequency of the green channel and the maximum frequency of the red channel are calculated as

3 $$\:{f}_{\text{x}-}^{G}={f}_{\text{x}+}^{G}-\frac{{x}_{+}-{x}_{-}}{{\lambda\:}_{G}{F}_{1}},$$

4 $$\:{f}_{\text{x}+}^{R}={f}_{\text{x}-}^{R}+\frac{{x}_{+}-{x}_{-}}{{\lambda\:}_{R}{F}_{1}}.$$

**Fig. 4:**
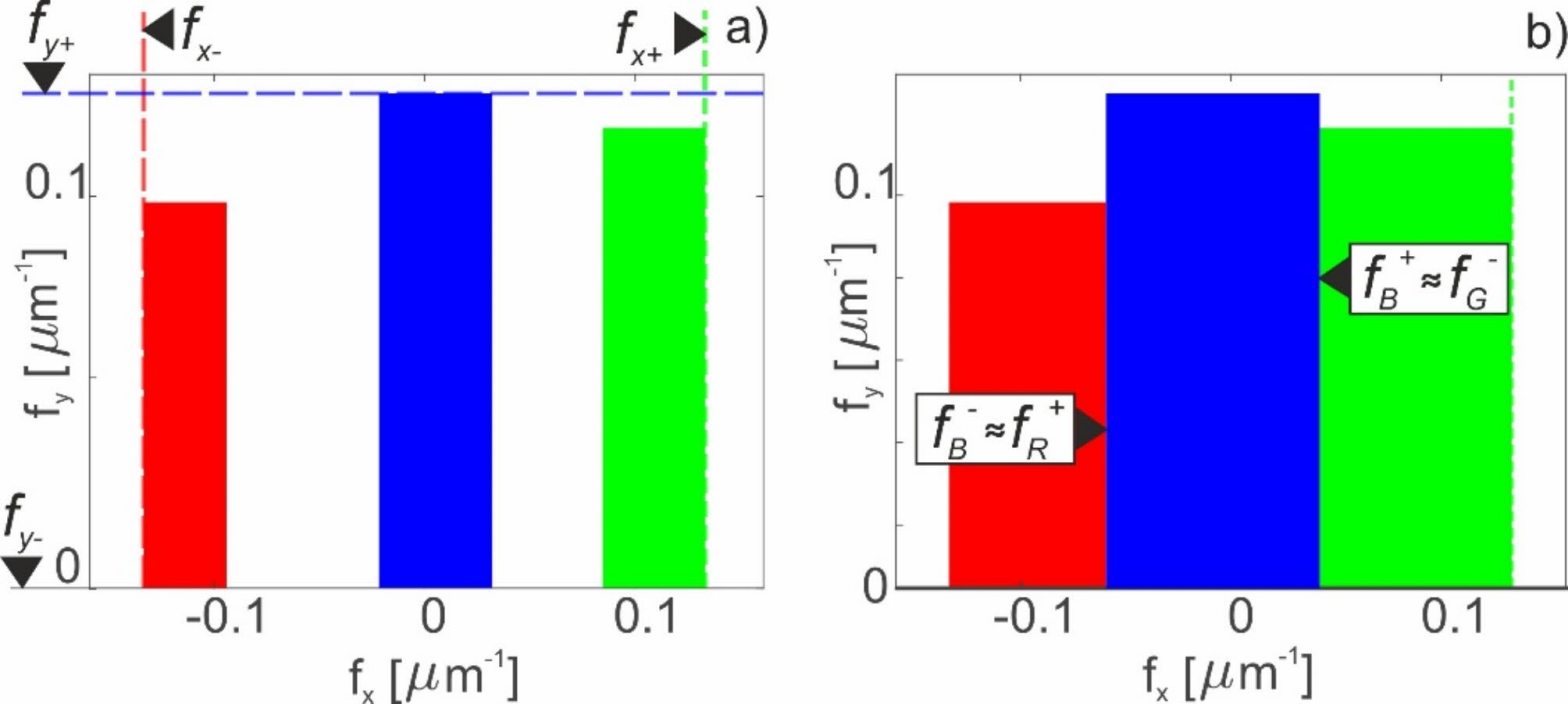
(**a**) Initial filter calculation for each color channel. (**b**) Final result for filter calculation.

These values are passed to the part of the algorithm illustrated on the right block of the diagram and RGB bands obtained at this step are illustrated in Fig. 4a. When selected frequencies fulfill the condition

5 $$\:{f}_{+}^{B}\approx\:{f}_{-}^{G},$$

6 $$\:{f}_{-}^{B}\approx\:{f}_{+}^{R}.$$

The frequencies of the RGB channels are found. Thus, the width of RGB channels is

7 $$\:\varDelta\:{f}_{c}=({f}_{+}^{c}-{f}_{-}^{c}),$$

where the index *c* indicates the corresponding color. Note that the algorithm determines the 4 F filter coordinates *x*_+_ and *x*_-_, which are found in the left block of the diagram. The filter bandwidth is then calculated based on these values. Thus, the bandwidth meets the condition $\:\varDelta\:{f}_{B}{\lambda\:}_{B}=\:\varDelta\:{f}_{R}{\lambda\:}_{R}=\varDelta\:{f}_{G}{\lambda\:}_{G}$. This feature of the algorithm allows increasing the speed of the hologram generation algorithm. Equations (1)–(4) enables calculating the frequencies of the light illumination of the RGB beams as

8 $$\:{f}_{xi}^{c}=\frac{{{l}_{B}f}_{x-}^{B}}{{l}_{c}}-{f}_{x-}^{c}\:.$$

The result of the algorithm is presented in the Fig. 4b, where obtained *f*_*x-*_^*B*^ = − 0.060 μm^−1^ and *f*_*x+*_^*B*^ = 0.0421 μm^−1^. Thus, for *x*-direction, the frequency limits of the RGB bands are (− 0.133 μm^−1^, − 0.059 μm^−1^) for red, (− 0.060 μm^−1^, 0.0421 μm^−1^) for blue, and (0.0422 μm^−1^, 0.133 μm^−1^) for green, respectively. If the condition is not met, the current x_+_ and x_−_ values are transferred to the part of the algorithm illustrated in the left block of the diagram, which starts the next calculation cycle.

In the display, the CGH is reconstructed with RGB light sources illuminating SLM from different angles defined by Eq. (8). Nevertheless, the spatial frequencies of the RGB channels encoding the color reconstruction of the 3D object must be aligned in the Fourier plane of the SLM. This means that the frequency bands for each color must occupy the same spatial region, which is bounded by the 4 F filter. The 4 F filter should have a size of (*x*_*+*_ − *x*_*−*_)×(*y*_*+*_ − *y*_*−*_). Therefore, to ensure the transfer of the full bandwidth of RGB component images, illumination angles and the position and size of the filter must be carefully adjusted.

For this, we propose a simple calibration procedure. The procedure starts with the design of an RGB Fourier hologram, in which the boundaries of three frequency filters, designed in previous section, are loaded. The complex Fourier hologram for each color is tailored as a super position of plane waves as follows

9 $$\:{\varPsi\:}^{c}=\left({\text{e}}^{i2\pi\:{f}_{\text{x}+}^{c}x}+{\text{e}}^{i2\pi\:{f}_{\text{x}-}^{c}x}\right)\sum\:_{m}{\text{e}}^{i2\pi\:{f}_{ym}^{c}y}+\left({\text{e}}^{i2\pi\:{f}_{\text{y}+}^{c}y}+{\text{e}}^{i2\pi\:{f}_{\text{y}-}^{c}y}\right)\sum\:_{n}{\text{e}}^{i2\pi\:{f}_{xn}^{c}x},$$

where *f*_*ym*_^*c*^ takes values from *f*_*y-*_^*c*^ to *f*_*y+*_^*c*^ and *f*_*xn*_^*c*^ takes values from *f*_*x-*_^*c*^ to *f*_*x+*_^*c*^. Note that the Ψ^*c*^ contains amplitude and phase information. Nevertheless, the phase reconstruction gives a well-seen two sets of vertical and horizontal lines.

**Fig. 5:**
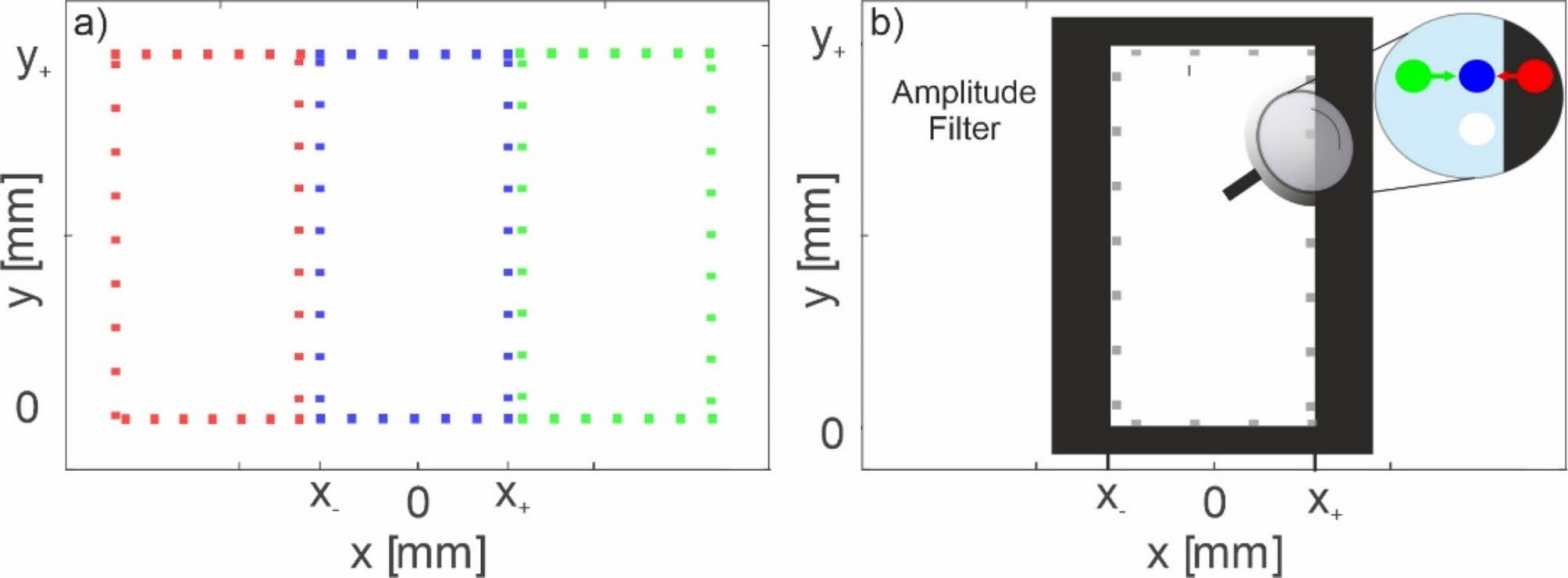
(**a**) Illustration of initial image formation at the back focal plane of lens L1. (**b**) Points superpositions after tilting red and green light sources.

The calibration procedure consists of two steps. In the first step, the position and size of the amplitude filter are set. For this purpose, the SLM displays the blue component of the CGH Ψ^B^, which is illuminated axially by a blue LED. As a result, two sets of vertical and horizontal lines appear in the Fourier plane, forming a square. The sides of this square correspond to the boundary frequencies *f*_*y-*_^*B*^, *f*_*y+*_^*B*^, *f*_*x-*_^*B*^, *f*_*x+*_^*B*^. A plane amplitude cut-off filter is then placed in the same plane, and its position and size are adjusted to ensure that all the corresponding information passes through the optical system. As a result, the 4 F filter has a size of (*x*_*+*_ − *x*_*−*_)×(*y*_*+*_ − *y*_*-*_). The second step of the calibration procedure ensures full bandwidth transfer for the red and green components. The corresponding illumination angles α for the green component and β for the red component are set to achieve this. All light sources are turned on, and the phases of and are loaded into the SLM. As a result, three sets of boundaries are generated in the Fourier plane, as illustrated in Fig. 5a. The green and the red beams are then adjusted by tilting them so that the boundaries of all three rectangles align, as shown in Fig. 5b. This calibration step also ensures that the power of each light source is balanced, providing equal energy across all channels, resulting in accurate white color points.

### FMCM CGH generation

**Fig. 6:**
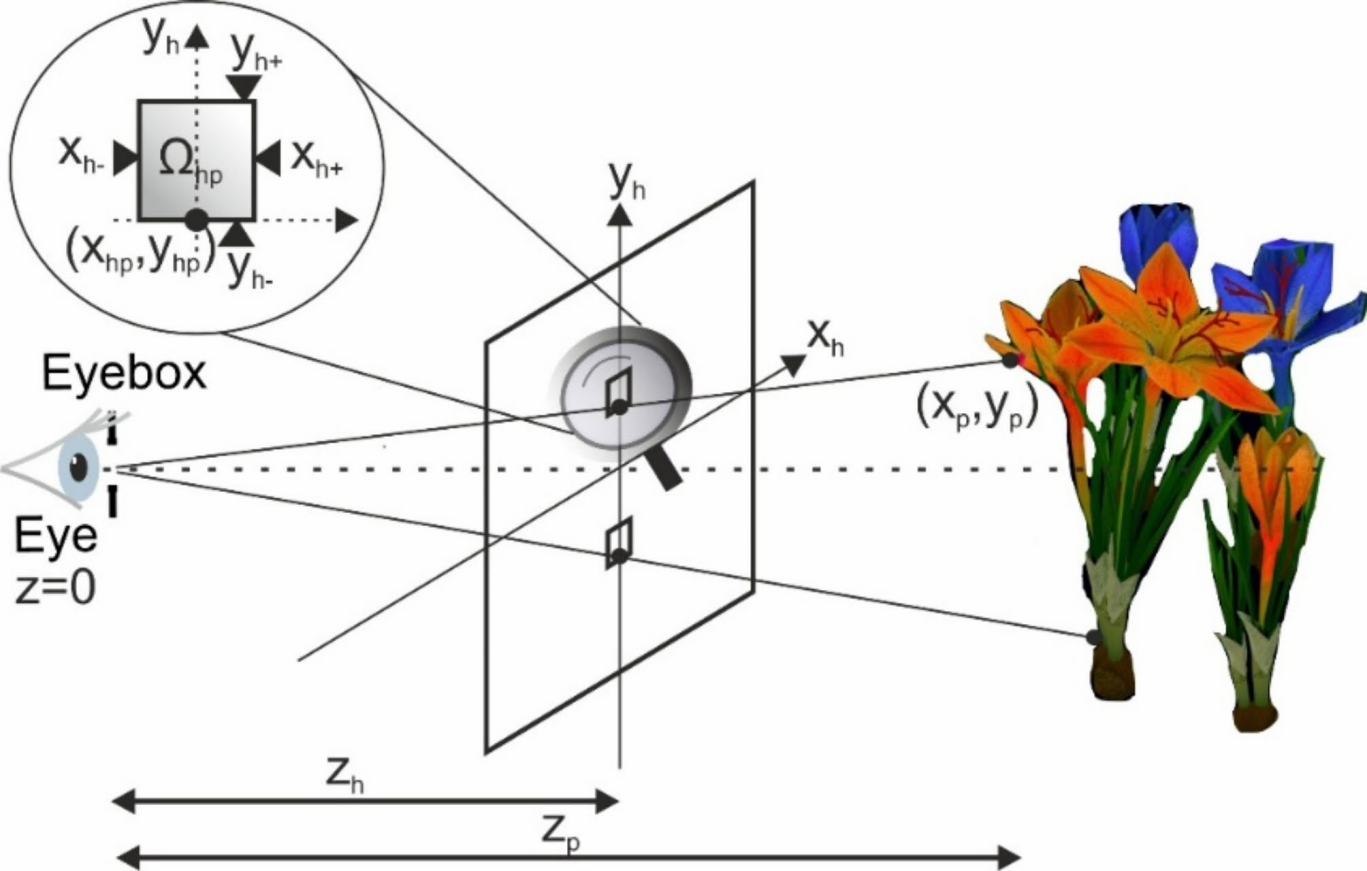
Geometry for CGH calculation.

The developed color CGH algorithm uses the concept of frequency multiplexing, where each RGB component occupies different frequency bands as shown in Fig. 4b. Thus, the color CGH denoted as *H* can be determined as the spatial sum of three RGB components as

10 $$\:H=\sum\:_{c}{H}_{c}=\sum\:_{c}{{R}_{c}^{*}U}_{c},$$

where *H*_*c*_, *U*_*c*_, and *R*_*c*_ are holograms, object waves, and reference waves, respectively. 3D CGH algorithms^32^ can be divided into point-cloud^33^, layer^34^, ray^35^, or polygonal^36^ methods. This work proposes a non-pupil WA-HNED system, in which 3D CGH can be calculated using only the point-cloud approach^7^. The CGH algorithm in this work, like all point cloud methods, calculates the hologram as the sum of P spherical waves encoding the object’s geometry. However, the algorithm of this work uses reference waves, which are spherical RGB waves with additional carrier plane waves for the R and G components. Thus, the color CGH can be computed as

11 $$\:H({x}_{h},{y}_{h})=\sum\:_{c}{e}^{-i{k}_{c}||{\mathbf{r}}_{\varvec{h}}||}{e}^{2\pi\:i{f}_{xi}^{c}{M}^{-1}{\text{x}}_{h}}\sum\:_{p}{a}_{p}^{c}{e}^{i{\text{F}}_{\varvec{p}}^{\varvec{c}}},$$

where

12 $$\:{\text{F}}_{\varvec{p}}^{\varvec{c}}={-\text{k}}_{c}||{\mathbf{r}}_{hp}^{c}-{\mathbf{r}}_{\text{p}}^{c}||\:\text{f}\text{o}\text{r}\:{\mathbf{r}}_{hp}^{c} {{\hat{I}}} {\varvec{\Omega\:}}_{hp}^{c}$$

and $\:{\mathbf{r}}_{\varvec{h}}=\left[{x}_{h},{y}_{h},{z}_{h}\right]$ is coordinate on the hologram plane as shown in Fig. 6 and $\:||\mathbf{r}||={\left({x}^{2}+{y}^{2}+{z}^{2}\right)}^{1/2}$, and *M* is a system magnification. Equation (11) is calculated for *p*∈1:*P* of the 3D geometry of the color object, which is represented by the coordinates vector $\:{\mathbf{r}}_{\text{p}}^{c}=[{x}_{p}^{c},{y}_{p}^{c},{z}_{p}^{c}]$ and the amplitudes $\:{a}_{p}^{c}$. The first two factors of Eq. (11)11 refer to the conjugates of RGB reference waves, while the sum for p refers to RGB object waves. Calculation of Eq. (11) is based on the principle of algorithm introduced in Ref.^7^, which is based on the fact that the optical wavefront information from the point source p on the object can be stored in a limited sub-hologram on the hologram plane. This region is sufficient to allow the full bandwidth of nonparaxial object information to be stored without aliasing. In addition, it is small compared to the size of the hologram. Thus, CGH generation requires processing a small amount of space bandwidth product ensuring efficient computation. Here CGH described by Eq. (11) for point *p* is calculated for the region $\:{\varvec{\Omega\:}}_{hp}^{c}$, which corresponds to the bandwidth of the 4 F filter. This bandwidth is different for every color and its range is

13 $$\:{f}_{y-}^{c}<{f}_{y}<{f}_{y+}^{c},$$

14 $$\:\frac{{x}_{-}}{{F}_{1}{l}_{c}}<{f}_{x}<\frac{{x}_{+}}{{F}_{1}{l}_{c}}.$$

For these frequency limits, according to the phase space analysis developed in Ref.^7^, the spatial limits of the sub-hologram $\:{\varvec{\Omega\:}}_{hp}^{c}\:$can be found as

15 $$\:{y}_{h-}^{c}<{y}_{h}<{y}_{h+}^{c},$$

16 $$\:{x}_{h-}^{c}<{x}_{h}<{x}_{h+}^{c},$$

where

17 $$\:{y}_{h\pm\:}^{c}=\frac{{f}_{y\pm\:}^{c}{\lambda\:}_{c}\left({z}_{o}-{z}_{hp}\right){||{r}_{hp}||}^{3}}{M\left({z}_{h}^{2}-{x}_{hp}^{2}\right){z}_{o}},$$

18 $$\:{x}_{h\pm\:}^{c}=\frac{({f}_{x\pm\:}^{c}+{f}_{xi}^{c}){\lambda\:}_{c}\left({z}_{o}-{z}_{hp}\right){||{r}_{hp}||}^{3}}{M\left({z}_{h}^{2}+{y}_{hp}^{2}\right){z}_{o}},$$

where $\:{\mathbf{r}}_{\mathbf{h}\mathbf{p}}=\left[{x}_{hp},{y}_{hp},{z}_{h}\right]=\left[{x}_{p}{z}_{h}{z}_{p}^{-1},{y}_{p}{z}_{h}{z}_{p}^{-1},{z}_{h}\right].$ Analyzing the Eqs. (17) and (18), it can be found that the spatial limits are equal for each component, and therefore

19 $$\:{\varvec{\Omega\:}}_{hp}={\varvec{\Omega\:}}_{hp}^{b}{\cong\:\varvec{\Omega\:}}_{hp}^{r}{\cong\:\:\varvec{\Omega\:}}_{hp}^{g}.$$

This relationship means that for a single point the components of the RGB hologram are calculated using the same sub-hologram size $\:{\varvec{\Omega\:}}_{hp}$. In this way, hologram calculations can be simplified and CGH performance can be improved. The algorithm flow of the FMCM algorithm is shown in Table 2.

**Table 2:**
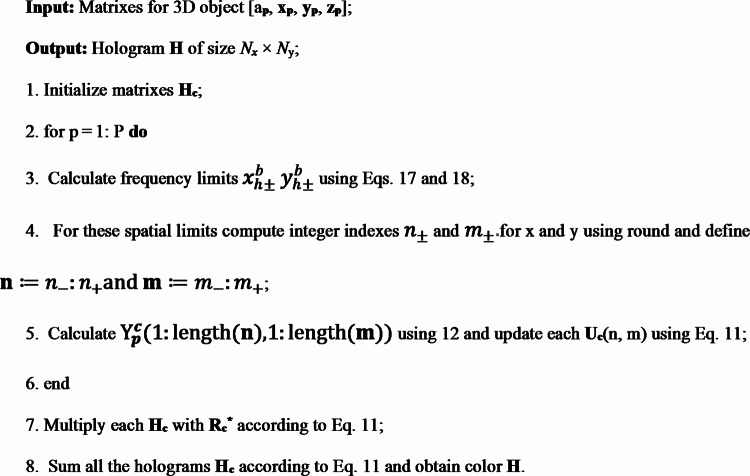
FMCM algorithm flow.

## Electronic supplementary material

Below is the link to the electronic supplementary material.

Supplementary Material 1

Supplementary Material 2

## Data Availability

Data sets generated during the current study are available from the corresponding author on reasonable request.
