# Supplementary material for: Wide-angle color holographic near eye display with full bandwidth frequency multiplexing
Source: Sci Rep. 2025 Apr 30;15:15221. doi: 10.1038/s41598-025-98411-3 (PMC12043964; doi:10.1038/s41598-025-98411-3)
Supplement: Supplementary file 2 — Supplementary Material 2 [file 41598_2025_98411_MOESM2_ESM.docx]

Description of Supplementary Video

**Title:** Supplementary Video 1

**Legend:** A sequence of 1140 frames, each showing different views of the rotating 3D color flowers displayed at the full-frame refresh rate of the SLM.
